# Increased synovial lipodystrophy induced by high fat diet aggravates synovitis in experimental osteoarthritis

**DOI:** 10.1186/s13075-017-1473-z

**Published:** 2017-12-01

**Authors:** Ane Larrañaga-Vera, Ana Lamuedra, Sandra Pérez-Baos, Ivan Prieto-Potin, Leticia Peña, Gabriel Herrero-Beaumont, Raquel Largo

**Affiliations:** 1 Bone and Joint Research Unit, IIS-Fundación Jiménez Díaz UAM, Avda. Reyes Católicos, 2, Madrid, 28040 Spain; 2 Clinical Analysis Department, HU-Fundación Jiménez Díaz, Madrid, Spain

**Keywords:** Osteoarthritis, Hypercholesterolemia, Synovial inflammation, Metabolic syndrome, Macrophages, Synovial adipose tissue, Adipokines

## Abstract

**Background:**

Metabolic syndrome (MetS) may be associated with knee osteoarthritis (OA), but the association between the individual components and OA are not well-understood. We aimed to study the effect of hypercholesterolemia on synovial inflammation in knee OA.

**Methods:**

OA was surgically induced in rabbits fed with standard diet (OA group, *n* = 10) or in rabbits fed with high fat diet (OA-HFD, *n* = 10). Healthy rabbits receiving standard diet (Control, *n* = 10) or fed with HFD (HFD, *n* = 6) were also monitored. Twelve weeks after OA induction, synovial membranes were isolated and processed for studies.

**Results:**

Animals fed HFD showed higher levels of total serum cholesterol, triglycerides and C-reactive protein than control rabbits. Twelve weeks after OA induction, synovial membrane inflammation and macrophage infiltration were increased in rabbits with OA, particularly in the OA-HFD group. Extensive decrease of synovial adipose tissue area, adipocyte size and perilipin-1A synthesis were observed in the OA-HFD group in comparison to the OA and control groups. The HFD further increased the proinflammatory mediators IL-1β, IL-6 and TNF in the OA synovium. However, the synovial gene expression of adipokines, such as leptin and adiponectin, were markedly decreased in the rabbits with OA, especially in the OA-HFD group, in correlation with adipose tissue loss. However, circulating leptin was upregulated in the HFD and OA-HFD groups.

**Conclusion:**

Our results indicate that a HFD is an aggravating factor worsening synovial membrane inflammation during OA, guided by increased infiltration of macrophages and removal of the adipose tissue, together with a remarkable presence of proinflammatory factors. Synovial adipocytes and dyslipemia could probably play pivotal roles in OA joint deterioration in patients with MetS, supporting that the link between obesity and OA transcends mechanical loading.

**Electronic supplementary material:**

The online version of this article (doi:10.1186/s13075-017-1473-z) contains supplementary material, which is available to authorized users.

## Background

Osteoarthritis (OA) is the most common joint disorder worldwide, characterized by joint pain, impaired mobility and structural changes in the joints. Although cartilage destruction is the main feature of the disease, every joint structure such as the synovium, bone, meniscus or muscle is affected, leading to the recognition of OA as a whole-organ disease [1]. Synovial inflammation is present in a substantial population of patients with OA and has been associated with different signs and symptoms of the disease, including increased pain and joint effusion, which could promote more rapid cartilage degeneration [2, 3]. Elevated thickness of the lining layer and greater presence and activation of synovial macrophages have been identified in cartilage degradation and osteophyte formation in both human and experimental OA [4–6].

OA is not merely a local disease, but there are different systemic processes that determine its progression. The concept of metabolic OA, coined in recent years, identifies a syndrome whereby the contribution of metabolic dysregulation and low-grade systemic inflammation to the progression of the disease has been firmly pointed out [7, 8]. The metabolic syndrome (MetS) comprises a cluster of conditions, including glucose intolerance, high blood pressure, hypercholesterolemia and hypertriglyceridemia, and obesity [9, 10]. The accumulation of the different components of MetS has been related to both the occurrence and progression of knee OA [11, 12]. However, little is known about the specific contribution of each of these metabolic alterations in OA progression, and specifically in the synovial damage associated with OA.

The contribution of obesity to OA progression is probably the most extensively studied association [7, 13]. In fact, obesity has been pointed out as the main contributing factor for the association between OA and MetS, in studies showing a markedly attenuated association after adjustment for body mass index [14]. However, OA is also common in non-weight bearing joints of obese persons, suggesting a systemic mechanism rather than a simply mechanic phenomenon [7].

The possible role of hyperlipidemia in mediating obesity-related effects on OA has been explored in different studies. Contradictory results have been published on the relationship between serum lipids and OA incidence in humans, probably due to the presence of obesity and being overweight as confounding factors [15]. In turn, different experimental studies have suggested that hypercholesterolemia could be mainly associated with osteophyte generation rather than to aggravation of cartilage lesions [16, 17]. Macrophages, endothelial cells and fibroblasts are dominant cells within the synovium, together with abundant adipose tissue that constitute the synovial stroma, and every component is sensitive to changes in lipid levels [17, 18].

Adipokines have been considered at least partially responsible for the link between systemic metabolic alterations and OA [19–21]. Adipokines are essentially released by adipocytes and exhibit pleiotropic functions both in central and peripheral systems, including blood pressure control, hemostasis, food intake, energy expenditure, cell metabolism and inflammation, among others [19–21]. They are also synthesized by joint cells during OA, mainly by the synovium, cartilage and intra-articular fat tissue, and have been demonstrated to play proinflammatory and catabolic or anabolic roles in OA pathophysiology. It has been hypothesized that the altered circulating patterns of adipokines induced by obesity could be responsible for the deleterious effect of this disease on OA. However, it is not known whether expression and release of adipokines in the joint could be modulated by metabolic factors during OA, thus contributing to disease progression.

Therefore, this work aimed to study the effect of hypercholesterolemia, without any other component of the MetS, on synovial inflammation in an experimental model of knee OA. We have also determined the synovial expression and systemic concentration of adiponectin and leptin, two adipokines involved in joint deterioration associated with metabolic OA.

## Methods

### Animal model

Thirty-six New Zealand male white rabbits, 13–15 weeks of age, weighing 2.5–3.0 kg (Granja San Bernardo, Navarra, Spain) were housed individually in cages with transparent walls (0.5 m cage height and 0.6 m^2^ floor space) exposed to a 12-hour light/dark cycle.

**Fig. 1:**
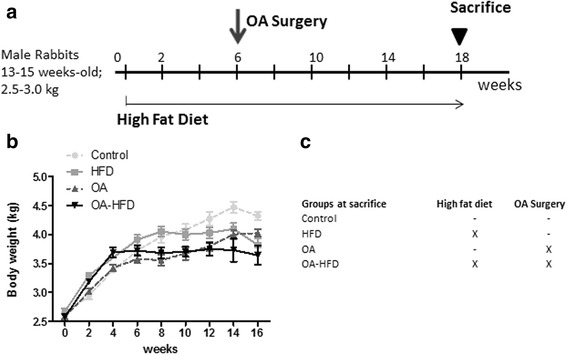
Animal model. **a** Schematic representation of the experimental model. *Arrow* indicates the time of osteoarthritis (OA) induction by surgical partial medial meniscectomy plus anterior cruciate ligament transection. *Arrowhead* indicates the end of the study, when all animals were killed and samples extracted. **b** Change in body weight during follow up. **c** Scheme of the interventions in each group studied. HFD, high-fat diet

After 2 weeks of adaptation to our facilities, 16 rabbits started receiving a high fat diet (HFD) (0.5% cholesterol + 4% peanut oil; S9504-S010; 22% kJ from fat, 20% kJ from proteins and 58% kJ from carbohydrates; Ssniff, Soest, Germany) administered *ad libitum* (Fig. 1a, time point 0). At this time point, there were no significant differences between the group on HFD and the one that remained at standard diet (112; 10% kJ from fat, 17% kJ from proteins; 73% kJ from carbohydrates; Safe-Diets, Augy, France) regarding body weight or age, as can be observed in Fig. 1b. Six weeks later, bilateral osteoarthritis (OA) was surgically induced in 10 of these 16 animals (OA-HFD group, *n* = 10) by anterior cruciate ligament transection and partial medial meniscectomy [22] (week 6, Fig. 1a). At this time point, OA was also induced in 10 rabbits fed with standard diet (OA group, *n* = 10). The surgery was always performed in the morning after overnight fasting, under general anesthesia (intramuscular administration 20 mg/ml xylazine (Rompun, Bayer, Kiel, Germany) and 50 mg/ml ketamine (Ketolar, Pfizer, Hameln, Germany) in a 3:1 ratio), under aseptic conditions in an operating room. Besides, 10 rabbits fed with standard diet (control group, *n* = 10), and six rabbits fed with the HFD (HFD group, n=6) underwent no experimental intervention.

Two animals in the OA-HFD group died during the time of the study due to OA surgery complications. Weight gain was monitored every week. Systolic blood pressure (SBP) was measured before OA surgery and 1 week before euthanasia, using a High Definition Oscillometry unit (DVM Solutions Houston TX, USA) adapted to the hind paw of the rabbits. This non-invasive method for SBP measurement has been validated in cats, an animal physiologically and anatomically similar to rabbits [23].

Twelve weeks after OA induction (Fig. 1), overnight-fasted rabbits were bled from their marginal ear vein in the morning and killed by an intracardiac injection of pentobarbital (50 mg/kg, Tiobarbital, Braun medical S.A. Barcelona, Spain). The articular cavity of each rabbit was accessed by sectioning the patellar tendon and taking out the patella, thus the entire infrapatellar synovial membrane (SM) was collected by the same operator (AL-V), always taking the same specimen from each animal (Additional file 1: Figure S1). The SM was not separated from the adipose tissue [24, 25]. Half of the SM containing both stroma and lining was then fixed in 4% paraformaldehyde for 24 h and then was embedded in paraffin for histological studies; the other portion was immediately frozen and used for molecular biology studies. Femoral condyles were also removed and fixed in 4% buffered paraformaldehyde, decalcified for 4 weeks in a solution of 10% formic acid plus 5% paraformaldehyde, and embedded in paraffin [26]. The left and right SM and condyles were analyzed as independent samples.

### Serum and synovial measurements

Glucose, total cholesterol, HDL cholesterol and triglyceride levels were assayed by automatic techniques as previously described [27, 28]. Adiponectin, leptin and plasma C-reactive protein (CRP) were measured by ELISA using commercial specific kits (SEA605Rb and SEA084Rb, respectively, USCN, Houston TX, USA and ab157726, Abcam, Cambridge, UK). Both adiponectin and leptin were measured in synovial tissue homogenates. For this purpose, total protein from the SM was extracted as described elsewhere [28, 29], and equal amounts of proteins diluted in the same volume for each knee were tested by specific ELISA for each adipokine.

### Histological synovitis grading

The SM from both knees of each rabbit were sectioned 5-μm thick and stained with hematoxylin and eosin, and Masson’s Trichrome. Synovitis was evaluated according to the Krenn score [30] as previously described [28], assessing lining hyperplasia, activation of synovial stroma related to fibrosis, and tissue infiltration. Each item was evaluated by a blinded observer using a subscale of 0− 3 points, where 0 indicated absence, 1 mild, 2 intermediate and 3 strong evidence of synovitis. The total score was obtained from the sum of partial grades with a maximum total score of 9.

### Histological cartilage grading

The decalcified femurs were cleaved in a sagittal plane along the central portion of the articular surface of each medial femoral condyle corresponding to the weight-bearing area, and subsequently embedded in paraffin wax. Cartilage was sectioned 5-μm thick and stained with hematoxylin/eosin and alcian blue to evaluate cartilage abnormalities. These samples were evaluated using a modified version of Mankin's grading score system, which analyses four different parameters with a total score up to 21: structure (0–8), proteoglycan staining (0–6), loss of chondrocytes (0–4), and clone formation (0–3) [25, 31].

### Immunohistochemical analysis

SM infiltrating macrophages were visualized using mouse anti-rabbit macrophage monoclonal antibodies (mAb) (RAM11; Dako, Glostrup, Denmark) as previously described [27], whereas adipocytes were identified with anti-perilipin A1 (PLIN, Abcam, ab61682, 1/100 dilution) antibody. To evaluate RAM11-positive immunoreactivity, five photographs were obtained using a Leica DMD108 digital micro-imaging instrument (Leica, Microsystems, Inc. Buffalo Grove, IL, USA) at × 10 magnification ensuring constant light exposure. Each image was analyzed with ImageJ software (NIH, Bethesda, MD, USA), and the percentage of positive area was calculated with the Color Deconvolution plugin [32] in relation to the total tissue area. For each SM, the percentage of positive staining was calculated as the mean of these five images corresponding to the same SM [28].

Adipose tissue area (%ATA) and adipocyte size were analyzed in PLIN-stained slides using the Coreo Iscan Au Scanner (Ventana Medical Systems, USA) and ImageJ software. Five representative images at × 20 magnification were used to identify stained adipocyte boundaries. Every white area showing no immunoreactivity to PLIN was manually removed. Finally, the area of each adipocyte was measured and the average size was calculated for each SM sample.

### Western blot

Briefly, total protein was extracted from the SM as described elsewhere [28, 29]. Protein extracts were separated by SDS-PAGE and transferred to a polyvinylidene fluoride membrane. The following primary antibodies were applied overnight at 4 °C: anti-human collagen type I (Col I, Merck Millipore, Billerica, MA, USA); anti-human PLIN (Abcam), anti-rabbit IL-1, anti-rabbit IL-6, anti-rabbit TNF (Cloud-Clone Corp, Houston TX, USA), and anti-human cyclooxygenase-2 (COX-2) (Santa Cruz Biotechnology, Dallas TX, USA). Loading control was performed employing EZBlue gel staining reagent (Sigma-Aldrich). Results were normalized relative to total protein presence and expressed as arbitrary densitometric units [28] (AU).

### Gene expression

Total RNA was extracted from SM using TriPure Isolation Reagent (Roche Diagnostics, Indianapolis, IN, USA), according to the manufacturer’s instructions. RNA was reverse-transcribed and RNA expression was quantified using the StepOnePlus™ detection system and StepOne™ software v2.2 (Applied Biosystems) as previously described [27, 33]. TaqMan® primers and probes were used to measure adiponectin (Oc03823307_s1), leptin (Oc03395809_s1) and Glyceraldehyde-3-phosphate dehydrogenase (GAPDH Oc03823402_g1) as endogenous control. Target genes were normalized relative to the expression of the endogenous control.

### Statistical analysis

Histological analyses were carried out by two observers (AL-V and RL) in a blinded fashion. Scoring and quantitative analyses were averaged for the images and sections from the same SM to calculate the value per sample for statistical analyses. Each limb was analyzed as an independent sample for the studies of synovial tissue. All statistical analyses were performed using GraphPad Prism version 5.0 for Windows (GraphPad Software, San Diego, CA, USA). We employed the non-parametric Kruskal-Wallis test with a post-hoc correction for (Dunn’s procedure) for comparisons between multiple groups, and the Mann-Whitney U test for comparisons between two groups. *P* values less than 0.05 were considered significant. Data are expressed as the mean ± 95% confidence interval (CI).

## Results

### Metabolic profile

We first studied the effect of the HFD in rabbits over an 18-week period in order to ensure the different characteristics that have been associated with MetS, such as being overweight, hypertension, basal glucose and dyslipidemia. There were no significant differences between the different groups in weight gain at week 6, the time point of surgery to induce OA (Fig. 1b). At the end of the study after 18 weeks of HFD feeding, rabbits fed a HFD gained less weight than controls (Table 1). Animals in the OA and OA-HFD groups also gained less weight than controls, probably due to discomfort associated with knee surgery. Rabbits in the HFD, OA and O-HFDA groups maintained similar SBP to control animals during the whole study period (Table 1). After 18 weeks of HFD, rabbits did not have any alteration in basal glucose levels or in oral glucose tolerance (data not shown) in comparison to control animals (Table 1). However, there was increased total serum cholesterol and triglycerides in the rabbits fed HFD in comparison to controls. Although no significant differences were observed in circulating CRP levels between either the HFD or OA-HFD groups and controls (Table 1), there was a significant increase in CRP in animals fed with HFD vs. those fed with the standard diet, as a result of grouping rabbits into HFD plus OA-HFD and control plus OA (27.1 ± 7.3 vs 10.5 ± 1.9, *p* = 0.026).

**Table 1:** Characterization of the rabbit model

Group	Weight gain (kg)	SBP (mmHg)	Basal glucose (mg/dl)	Cholesterol (mg/dl)	Triglycerides (mg/dl)	HDL (mg/dl)	CRP (μg/ml)
Control (*n* = 10)	1.9 (1.7–2.0)	100 (93–107)	109 (101–116)	32.2 (8.9–55.5)	48 (32–64)	11.1 (8.6–13.6)	15.95 (8.2–23.7)
HFD (*n* = 6)	1.5* (1.1–1.7)	103 (87–119)	105 (98–112)	1876* (1139–2613)	253* (4–502)	12.5 (6.9–18.1)	39.14 (1–77.3)
OA (*n* = 9)	1.5* (1.2–1.8)	108 (96–119)	105 (94–115)	28.3 (18.2–38.4)	67.3 (40–95)	13.8 (10.2–17.3)	6.9 (3.8–10.0)
OA-HFD (*n* = 8)	0.7* (-0.12–1.6)	106 (96–117)	104 (98–111)	2050* (1587–2514)	290* (99–480)	15.6 (11.0–20.3)	18.1 (5.5–30.7)

Measures were obtained from serum or plasma samples taken just before animals were killed. Values represent mean with 95% confidence interval

*HFD* high-fat diet, *OA* osteoarthritis, *SBP* systolic blood pressure, *HDL* high-density lipoprotein, *CRP* C-reactive protein

*P < 0.05 vs. Control

### Histological synovial inflammation and cartilage damage

**Fig. 2:**
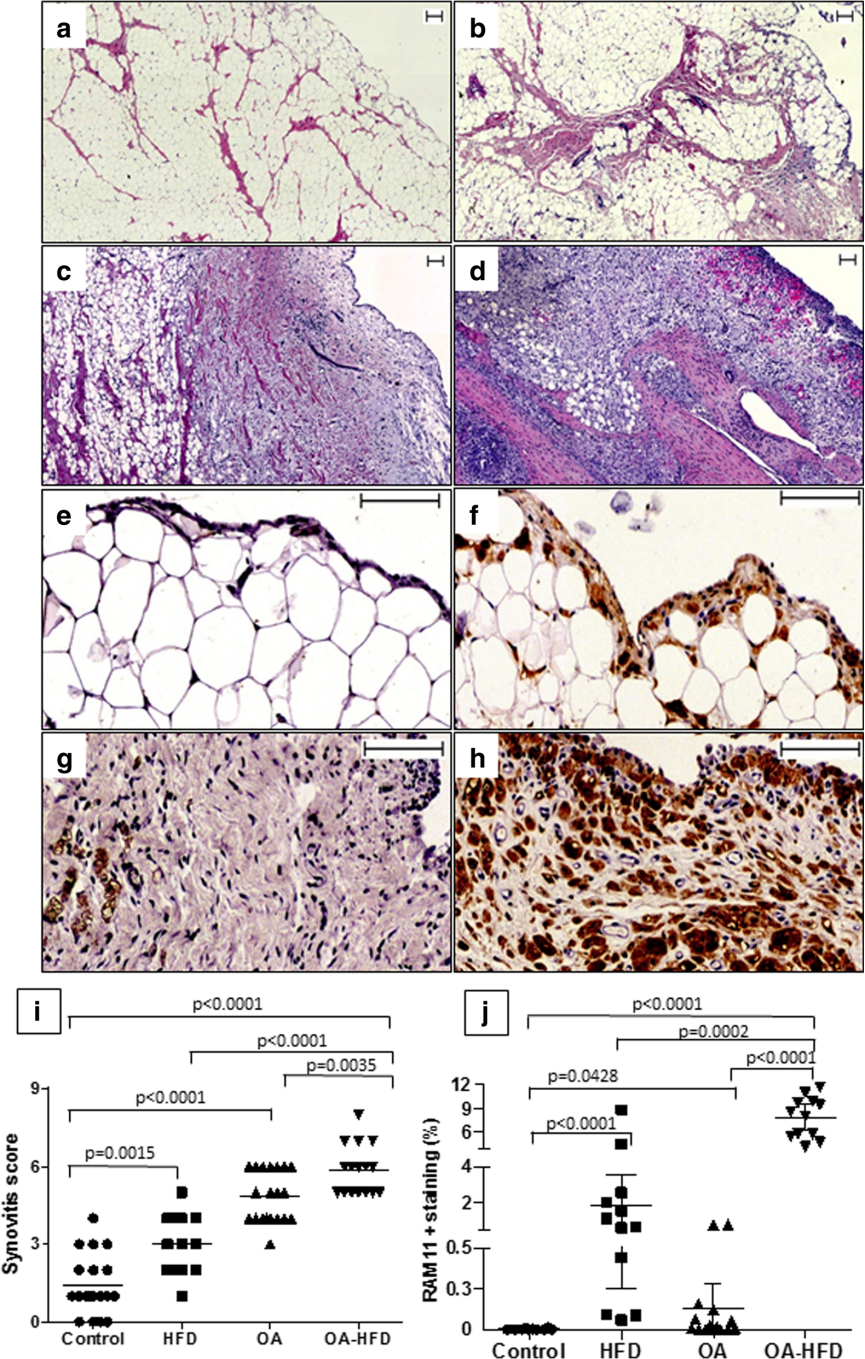
Histopathological and macrophage analysis in the synovial membrane (SM). **a**-**d** Representative sections of SM stained with hematoxylin-eosin or **e**-**h** stained with a monoclonal anti-rabbit macrophage antibody (RAM11) from Control rabbit (**a** and **e**); rabbit fed with a high-fat diet (HFD) (**b** and **f**); osteoarthritic (OA) rabbit (**c** and **g**); and OA rabbit fed with a HFD (OA-HFD) (**d** and **h**). **a**-**d** Scale bar = 100 μm. **e**-**f** Scale bar = 100 μm. **i** Synovitis score quantified as described in “Methods. **j** Quantification of RAM11-positive area represented as percentage of total area. Data from individual measurements and mean for each group are shown. *n* = 12–20 SM per group for histopathological analysis; *n* = 10–16 SM per group for RAM11 analysis

Rabbits fed HFD had mild lining hyperplasia, discrete presence of infiltrating cells and a slight increment in stromal fibrosis, and thus the synovitis score was significantly higher than that observed in healthy controls (Fig. 2b, 2i). The OA group had a higher synovitis score than the control and HFD groups, with similar lesions to those described in synovitis in humans with advanced OA: mild to moderate lining hyperplasia, discrete presence of inflammatory cells, and stromal activation. The OA-HFD group had mild lining thickening, a clear increment in stromal cellularity, presence of infiltrating cells and inflammatory foci. All samples had enlarged stroma with an intense cell density (Fig. 2d). The synovitis score in the OA-HFD group was significantly higher than in the other groups (Fig.2i).

The HFD administration did not modify the histological appearances of cartilage damage, with the HFD group having a similar score to control animals (HFD 2.8 ± 1.5 vs. control 2.9 ± 1.0; *p* not significant (NS)). In addition, HFD did not significantly modify the histopathological damage in the cartilage in the OA-HDF group in comparison to the damage observed in the OA group (OA 15.2 ± 2.1 vs. OA-HFD 14.0 ± 2.7; *p* NS).

### Macrophage infiltration and presence of foam cells

There was moderate presence of macrophages in the SM of rabbits fed HFD, which were especially localized in the lining layer (Fig. 2f, j). Lipid droplets were identified in their cytoplasm and their morphological shape resembled to pro-atherosclerotic foam cells, as previously described [5, 28] (Fig. 2f). RAM11 staining was scarce in the SM in the OA group, whereas there was extensive infiltration of RAM11-positive cells in the OA-HFD group to a much greater extent than in the HFD and OA groups (Fig. 2g, h, j). They were both consistently localized in the lining and sub-lining layers in every sample, and had the characteristic phenotype of foam cells [5, 28] (Fig. 2h).

### Characterization of synovial stroma

**Fig. 3:**
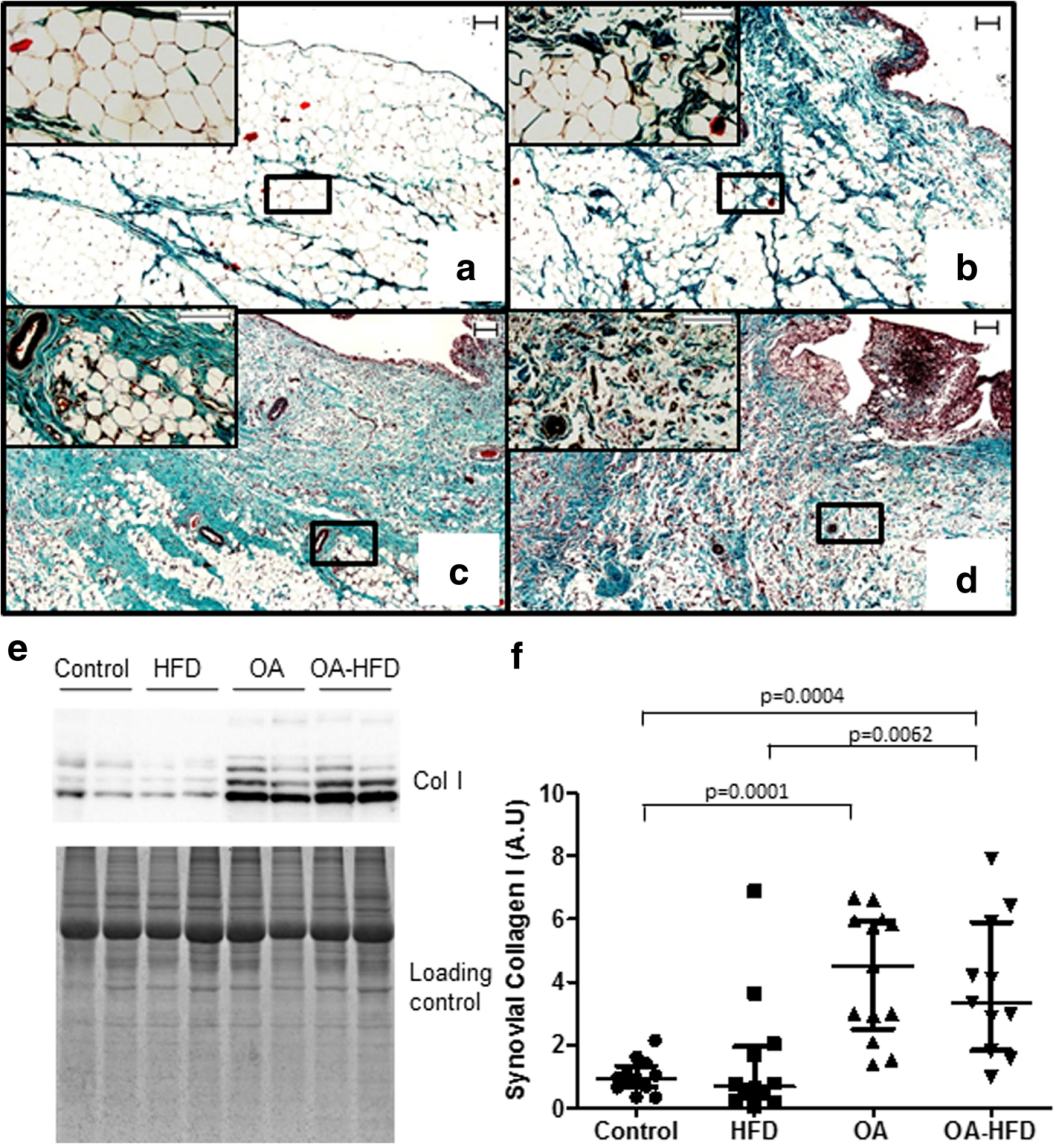
Characterization of synovial stroma. Representative sections of synovial membranes stained with Masson's Trichrome. Magnification of the selected area is also shown. **a** Control healthy rabbit; **b**, rabbit on a high-fat diet (HFD); **c** osteoarthritic (OA) rabbit; **d** OA rabbit fed with HFD (OA-HFD); scale bar = 100 μm. **e** Representative western blot of collagen I (Col I) in the synovial membrane (SM) of the rabbits. EZ blue staining was used as protein loading control. **f** Densitometric analysis of Col I measured by western blot. Results are expressed as fold induction. Data from individual measurements and mean and 95% CI are shown (*n* = 10–14 SM per group)

Whereas healthy SM was mainly composed of adipocytes with little surrounding matrix, we observed patchy distribution of some fibrotic areas in the HFD group (Fig. 3a, b). OA membranes had a highly vascularized fibrotic stroma with some lax and dense stents, green-colored on Masson’s Trichrome staining (Fig. 3). OA-HFD samples also had highly vascularized fibrotic membranes. The quantification of col I protein revealed a clear increase in the fibrotic content of the SM in the OA and OA-HFD groups (Fig. 3e-f) in comparison to control and HFD groups.

### Adipose tissue area and adipocyte size in the SM

**Fig. 4:**
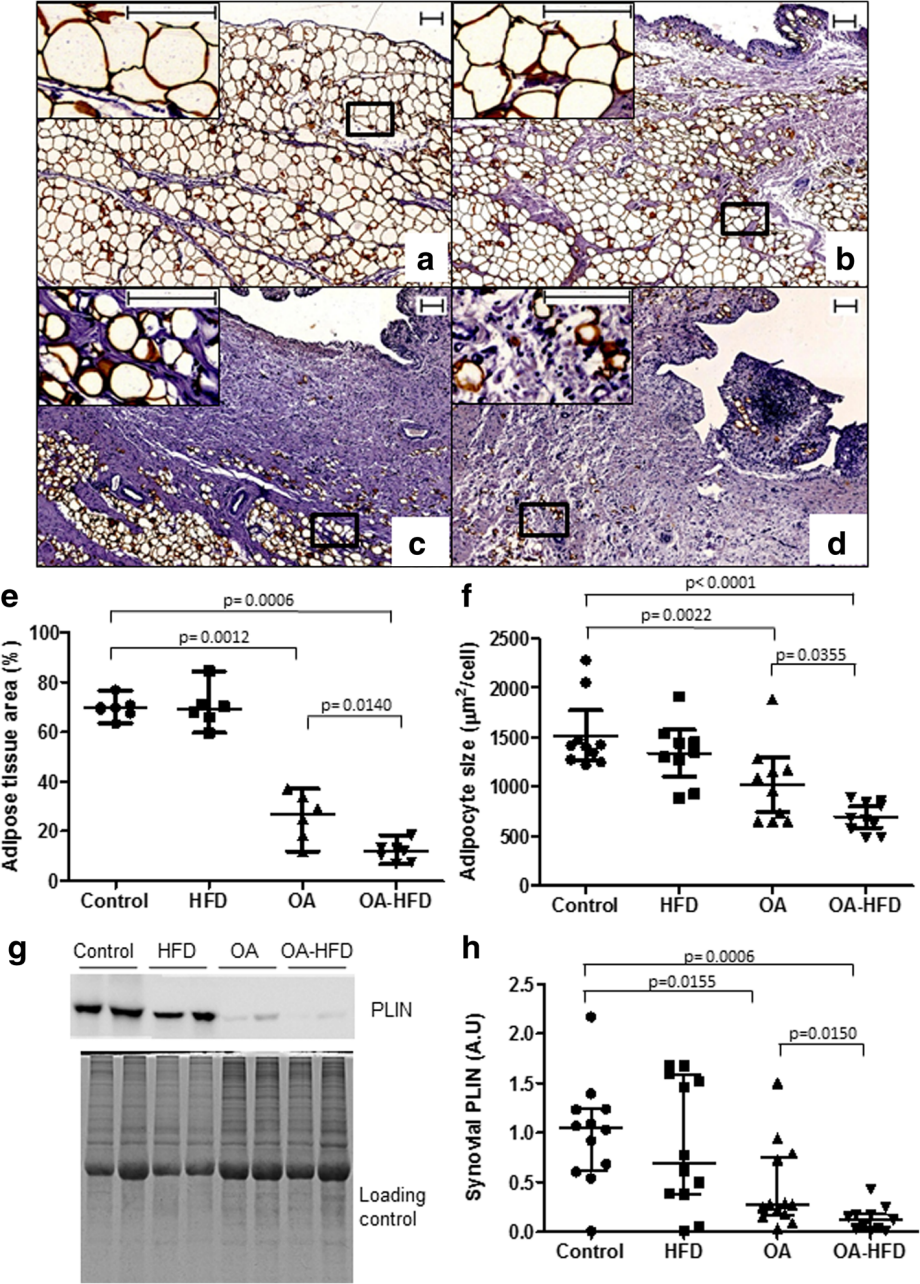
Characterization of adipose tissue area and adipocyte size. Representative sections of synovial membranes (SM) stained with a monoclonal perilipin 1A (PLIN) antibody. Magnification of the selected area is also shown. **a** Control healthy rabbit; **b** rabbit fed a high-fat diet (HFD); **c** osteoarthritic (OA) rabbit; **d** OA rabbit fed with HFD (OA-HFD); scale bar = 100 μm. **e** Percentage of adipose tissue area in the SM of each group. **f** Mean of the adipocyte size in the SM of each group. **g** Representative western blot of PLIN protein levels expressed in the SM. EZ *blue* staining was used as protein loading control. **h** Densitometric analysis of PLIN measurement by western blot expressed as fold induction. Data from individual measurements and the mean and 95% CI are shown (*n* = 9 − 14 SM per group)

We quantified the adipose tissue fraction using PLIN staining, a distinguishing marker of adipocytes [34, 35]. A clear diminution in the percentage of adipose tissue area (%ATA) in the SM in the OA and OA-HFD samples in comparison to control and OA groups was observed, which was even lower in the OA-HFD than in the OA group (Fig 4a-e). Furthermore, SM adipocytes were significantly smaller in both the OA and OA-HFD groups than in the controls (Fig. 4f). The shape of these cells in control tissues was regular (Fig. 4a), whereas we observed high heterogeneity in the appearance of these cells in the SM in the OA and OA-HFD groups (Fig. 4c, d). Adipocyte size further decreased in the SM in the OA-HFD group in comparison to the OA group (Fig. 4f). PLIN content in the SM was also evaluated by western blot. In correlation with the %ATA, there was diminution in the SM PLIN in the OA and OA-HFD groups in comparison to the controls (Fig. 4g, h). Of note, the synthesis of PLIN was also significantly diminished in the OA-HFD group in comparison to the OA group.

### Adipokine gene expression and concentration in SM and serum

**Fig. 5:**
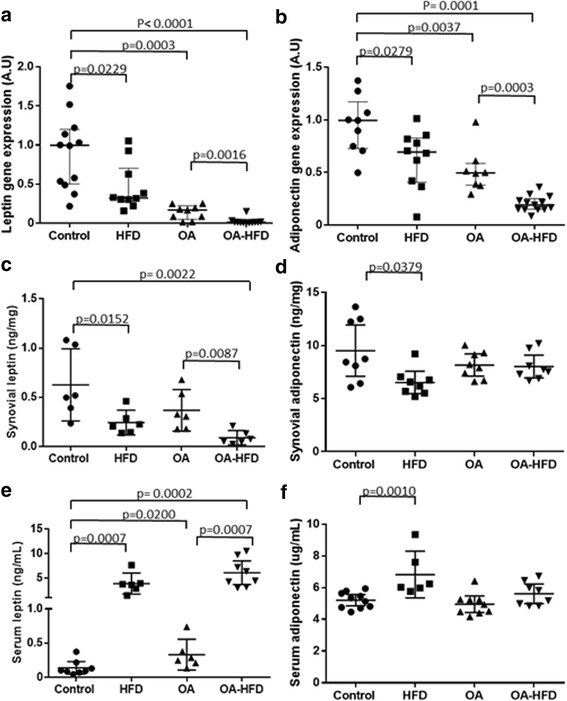
Adipokine expression profile in the synovial membrane (SM) and serum concentration. **a** Leptin gene expression in rabbit SM measured by quantitative (q)PCR (*n* = 10–14 SM per group). **b** Adiponectin gene expression in rabbit SM measured by qPCR (*n* = 10–14 SM per group). **c** Leptin protein expression in SM membrane measured by ELISA (*n* = 6 SM per group). **d** Adiponectin protein expression in rabbit SM measured by ELISA (*n* = 8 SM per group). **e** Leptin concentration in rabbit serum measured by ELISA (*n* = 6–10 rabbits per group). **f** Adiponectin concentration in rabbit serum measured by ELISA (*n* = 6–10 rabbits per group). Data from individual measurements and mean and 95% CI for each group are shown. HFD, high-fat diet; OA, osteoarthritis

Rabbits in both the HFD and OA groups had a clear decrease in leptin and adiponectin gene expression in the SM in comparison to controls (Fig. 5a, b). We observed an additive effect of these interventions in the OA-HFD group, where the gene expression of both leptin and adiponectin was significantly lower to that observed in the HFD and OA groups (Fig. 5). Interestingly, there was significant correlation between adipokine gene expression and the %ATA (*R* = 0.746; *p* = 0.001 for leptin expression; *R* = 0.732; *p* = 0.002 for adiponectin expression). Leptin levels in the SM measured by ELISA were decreased in the HFD group in comparison to controls, and it was also significantly reduced in the SM in the OA-HFD group in comparison to controls, the HDF and the OA groups (Fig. 5c). Adiponectin concentration only significantly diminished in the HFD group in comparison to controls (Fig. 5d). There was no correlation between the presence of these proteins and the %ATA in the SM.

However, HFD increased the circulating concentration of both adipokines, and there were no significant differences between the HFD and OA-HFD group in the serum concentration of these mediators (Fig. 5e, f).

### Synovial proinflammatory mediators

**Fig. 6:**
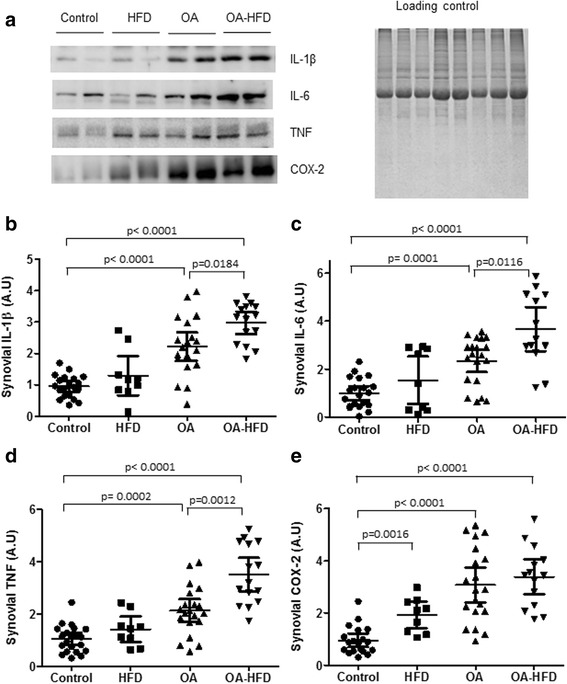
Proinflammatory mediators in the synovial membrane (SM). **a** Representative western blot of IL-1β, IL-6, TNF and cyclooxygenase-2 (COX-2) protein levels expressed in the SM of the rabbits in each group. EZ *blue* staining was used as protein loading control. **b**-**e** Densitometric analysis of IL-1β, IL-6, TNF and COX-2 measured by western blot. Data from individual measurements, expressed as fold induction and mean and 95% CI for each group are shown (*n* = 12–20 SM per group). HFD, high-fat diet; OA, osteoarthritis

We then explored whether the HFD was able to modify the presence of different proinflammatory cytokines, such as IL-1β, IL-6, TNF and COX-2 in the SM of rabbits in the OA group. As expected, western blot studies that OA induced a marked increase in the presence of all the studied proinflammatory mediators in comparison to control animals. The presence of hyperlipidemia further increased the presence of IL-1 β, IL-6 and TNF in the SM in the OA-HFD group in comparison to the OA group (Fig. 6).

## Discussion

In this study, we have shown that HFD aggravated OA synovitis, by inducing severe tissue architecture disorganization of the synovium, along with remarkable intensification of the proinflammatory cytokines IL-1β, IL-6 and TNF, and extensive infiltration of macrophages. However, HFD did not have any effect on the aggravation of the pathologic change in cartilage associated with OA. A relevant histological synovial alteration was the significant loss of synovial adipose tissue content, in correlation with decreased leptin and adiponectin gene expression.

In order to isolate the effect of hyperlipidemia, we employed an experimental model of HFD intake that was not associated with weight gain [28]. The lack of significant weight gain in the HFD group has been previously reported and attributed to the animal self-regulation of caloric intake [36]. In fact, animals in both the HFD and OA-HFD groups had a significant decrease in weight gain, which was probably related to the increase in systemic inflammation induced by the diet [28]. Different studies using lipid-rich diets have not been able to adequately apportion the contribution of added mechanical load and hyperlipidemia in OA, a factor that was avoided in our experiments.

Different patterns of synoviopathy have been described in patients with OA, both in late and early disease, such as those with an increased fibrotic component or those essentially characterized by augmented inflammatory parameters [37]. In our rabbits, OA synovitis was associated with a significant increment of fibrotic tissue and partial loss of adipose tissue, and scarce presence of macrophages. A HFD induced both qualitative and quantitative changes in the SM in the rabbits with OA. However, HFD did not significantly aggravate cartilage damage in either the HFD group or the OA-HFD group. These data are in line with previously published results [38, 39], and suggest that the aggravation in synovial inflammation induced by HFD is not a secondary event induced by more severe pathological change in the cartilage.

The higher grade of synovial inflammation in the OA-HFD group was characterized by the remodeling of adipose tissue and by adipocyte loss. The remaining adipocytes had heterogeneous morphology and were significantly smaller in comparison to the OA and control groups, confirmed by the decreased presence of PLIN. HFD further increased synovial macrophages, most of them with the appearance of foam cells, whereas the fibrotic component was similar to that observed in the OA group. To our knowledge, this is the first report of correlation between synovial inflammation and loss of adipose tissue in this localization. Contradictory reports have been published on the contribution of the volume or area of the intra-articular fat tissue to joint deterioration and OA symptoms [40–42]. However, the alterations in adipocyte size, morphology, loss of adipose tissue with increased fibrosis and inflammatory content have been well-described in inflamed adipose tissue in other anatomic localizations, and described as lipodystrophy [43, 44]. The study of the synovial fat pad as an independent adipose intra-articular tissue has contributed to its identification as a crucial player in OA progression [45], although lack of recognition of the synovium as a whole, integrated, functional and structural unit hampers the understanding of the mechanisms involved in the synovial alterations in OA. Histologically, synovial lining, adipose sub-lining and synovial fat pad represent a continuum. Sub-lining adipose tissue and the fat pad seem to share a common inflammatory state, both in cell content and cell phenotype, induced by the disease process more than by tissue-specific signals [24, 46].

The mechanisms by which adipose tissue can be replaced by fibrotic tissue in the OA synovium have not been fully elucidated. However, the increase in the hypoxia-associated mediators, induced by biomechanical alterations and proinflammatory cytokines, could be at least partially responsible for this phenomenon. An increase in hypoxia-induced factor-1 (HIF-1)α has been described in the OA synovium, in correlation with greater joint destruction [47, 48]. In adipose tissue, with a similar structure and cellular component to that observed in the stroma of the SM, HIF-1α induces tissue fibrosis and inhibits pre-adipocyte differentiation [49]. Furthermore, in inflamed adipose tissue from mice fed a HFD, HIF-1α-stimulated macrophages form highly hypoxic structures called crown-like structures (CLS), comprising macrophages encircling dead or dying adipocytes [50]. Indeed, we have previously identified CLS in the OA synovium in both human and hypercholesterolemic rabbits with synovial inflammation [5, 28].

Hyperlipidemia in rabbits in the OA group did not seem to enhance the presence of fibrosis-associated proteins, such as col I. However, it evoked a dramatic increase in macrophage infiltration in the synovium and greater decrease in adipose tissue content. Dyslipemia has been directly related to macrophage infiltration and inflammation in the synovium and adipose tissue [51]. In hyperlipidemic mice with OA, the synovial proinflammatory macrophage subset was identified as responsible for an increase in TNF synthesis and extracellular matrix remodeling in the synovial membrane [51]. In line with these data, we identified greater TNF expression in the synovium in the OA-HFD group that paralleled the increased macrophage density in this tissue. Although little is known about the metabolic regulation of synovial macrophages, prolonged lipid exposure could result in failure of the lipid-handling mechanisms, leading to different lipotoxic events, such as those described in obesity-associated insulin resistance, atherosclerosis and other inflammatory diseases related to MetS [52]. Thus, hyperlipidemia could drive M1 macrophage polarization in the OA synovium, resulting in a major presence of proinflammatory cytokines, as has been described in adipose tissue [52, 53]. Furthermore, adipocyte apoptosis and impaired adipogenesis have been also associated with the increased lipolysis induced by over-nutrition or HFD feeding [52]. Although hyperlipidemia could aggravate OA synovial inflammation, increasing macrophage density and adipose tissue destruction, the presence of hyperlipidemia per se could only have limited effects on SM alterations, as recently reported in HFD-fed mice [54].

Leptin and adiponectin gene expression diminished in the SM in the OA and OA-HFD group in comparison to control animals. These results appear to correlate with the amount of intra-articular adipose tissue rather than with the presence of a proinflammatory milieu. Furthermore, circulating leptin was significantly increased in HDF-fed animals, probably due to the effect of the diet on the extra-articular fat tissue [55]. Our data are in line with previous reports indicating that hyperlipidemia could be an aggravating factor for OA through the stimulation of systemic proinflammatory mediators [56]. In the OA group we also found increased circulating leptin as previously described in human and experimental OA, related to joint damage [19]. Therefore, our data do not support the hypothesis that hyperlipidemia could be an aggravating factor in metabolic OA, stimulating adipokine expression within the intra-articular adipose tissue. Different joint cells, such as chondrocytes or bone cells, could be responsible for adipokine synthesis in response to biomechanical or proinflammatory stimuli [19, 20].

## Conclusions

In summary, these data show that HFD aggravates the inflammation in the SM of rabbits with OA by inducing an increase in the infiltrating macrophages in the synovium, together with macrophage and metabolic-mediated remodeling of adipose tissue, and further elevation of proinflammatory cytokines. The lipotoxic effects induced by dyslipemia in adipocytes and macrophages could play a decisive role in the joint deterioration of patients with OA and MetS, supporting the hypothesis of a plausible link between obesity and OA going beyond mechanical loading.

## Additional file

Additional file 1: Figure S1. Synovial tissue collection. Photographs show the precise locations where the rabbit joint was accessed and the synovial membrane (SM) was collected. Half of the SM containing both stroma and lining was then fixed and embedded in paraffin for histological studies, and the other portion was immediately frozen and used for molecular biology studies. (TIF 376 kb)

## Abbreviations

ATA: Adipose tissue area
CLS: Crown-like structures
Col I: Collagen I
COX-2: Cyclooxygenase-2
CRP: C-reactive protein
ELISA: Enzyme-linked immunosorbent assay
HFD: High fat diet
IL-1β: Interleukin-1β
IL-6: Interleukin-6
MetS: Metabolic syndrome
OA: Osteoarthritis
PLIN: Perilipin-1A
SBP: Systolic blood pressure
SM: Synovial membrane
TNF: Tumor necrosis factor
