# Supplementary figures and images for: Increased synovial lipodystrophy induced by high fat diet aggravates synovitis in experimental osteoarthritis
Source: Arthritis Res Ther. 2017 Dec 1;19:264. doi: 10.1186/s13075-017-1473-z (PMC5709929; doi:10.1186/s13075-017-1473-z)

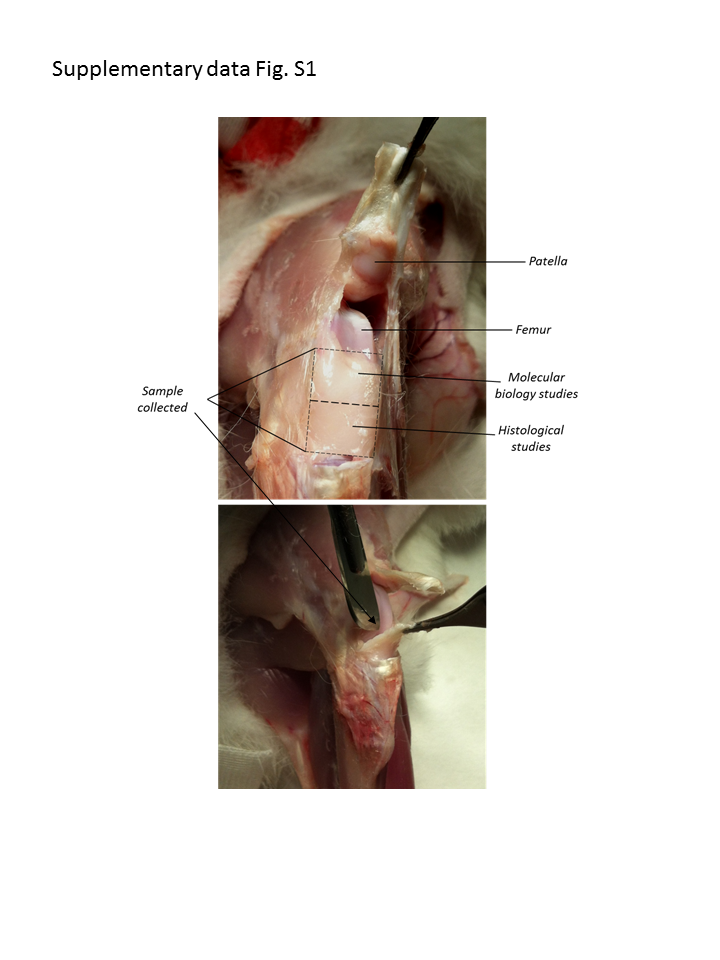

Supplement: Additional file 1: Figure S1. — Synovial tissue collection. Photographs show the precise locations where the rabbit joint was accessed and the synovial membrane (SM) was collected. Half of the SM containing both stroma and lining was then fixed and embedded in paraffin for histological studies, and the other portion was immediately frozen and used for molecular biology studies. (TIF 376 kb) [file 13075_2017_1473_MOESM1_ESM.tif]
